# Integrative Approaches in Pediatric Palliative Care

**DOI:** 10.3390/children5060075

**Published:** 2018-06-13

**Authors:** Kate Shafto, Suzanne Gouda, Kris Catrine, Melanie L. Brown

**Affiliations:** 1 Department of Internal Medicine and Pediatrics, University of Minnesota, Minneapolis, MN 55455, USA; kshafto@umn.edu; 2 Department of Pediatrics, University of Chicago, Chicago, IL 60637, USA; suzanne.gouda@uchospitals.edu; 3 Department of Pain Medicine, Palliative Care and Integrative Medicine, Children’s Hospitals and Clinics of Minnesota, Minneapolis, MN 55404, USA; kris.catrine@childrensmn.org; 4 Department of Pediatrics, University of Minnesota, Minneapolis, MN 55455, USA

**Keywords:** pediatrics, palliative care, integrative medicine, symptom management, mind-body medicine, pain management, acupuncture, yoga

## Abstract

Pediatric palliative care is a field which focuses on caring for and treating the symptoms and distress typically associated with life-limiting illness. Integrative medicine is supported by evidence and aims to heal the whole person, including all aspects of one’s lifestyle. Therapies offered by integrative medicine often empower patients and families, allowing for a sense of control. This review addresses the merging of integrative medicine philosophy and modalities with the care given to children with life-limiting illness. We review an introduction to integrative medicine, trends in its incorporation in the healthcare setting, application to patients receiving palliative care and the management of specific symptoms. A case study is offered to illustrate these principles.

## 1. Introduction: Integrative Medicine

Integrative medicine is not a new concept, though in recent decades the terminology has become solidified and more widely used. Dating back to early human history, there are writings describing how humans are composed of body, mind and spirit, with healers addressing these realms together when illness occurred [1]. The current definitions of integrative medicine center around a goal of healing, the patient’s lifestyle, environment, history, and a patient-provider partnership which considers lifestyle, allopathic diagnosis and treatment, environmental factors, emotional/spiritual factors and the role of other healing systems when caring for the patient. The University of Arizona’s Center for Integrative Medicine, USA, a leader in training and research around integrative medicine for several decades, defines integrative medicine as: “healing-oriented medicine that takes account of the whole person, including all aspects of lifestyle. It emphasizes the therapeutic relationship between practitioner and patient, is informed by evidence, and makes use of all appropriate therapies” [2].

Integrative medicine is distinct from complementary and alternative medicine (CAM), which refers to a practice or treatment that differs from the conventional approach, or therapies which complement the conventional model. Integrative medicine, as its name suggests, intentionally integrates too-often separate approaches to healing, with an additional emphasis on empowering the patient with skills for self-care, promotion of wellbeing and self-management (at times with even prevention or reversal) of disease. 

## 2. Integrative Medicine Trends in US Healthcare

Modern western medicine is based on a reductionist approach to the body and disease, dating back to the 1600s, out of which grew the current system of medical specialization and compartmentalization of organs and body systems [1]. This model has come at a great cost financially, and, in many cases, led to disempowerment of patients, a fragmented healthcare system, and patient dependence on healthcare providers for treatment and management of disease. Chronic diseases, due to their multi-organ pathophysiology and high correlation with lifestyle, are often not adequately addressed using a reductionist model. Additionally, the locus of control in an allopathic model is frequently external to the patient, being the healthcare provider and their prescribed treatment. A study examining the health locus of control for youths with chronic illnesses found that those with an external locus of control had poorer health outcomes than those with an internal locus of control [3]. Integrative medicine seeks to leverage a combination between the internal and external, which in turn shifts the locus of control to include a patient’s own capacity to influence their health or disease in addition to the provider.

There is clear and rising interest among patients as well as providers in a whole-person care model. Fellowships in integrative medicine are providing evidence-informed knowledge and tools for practice [4]; additionally, a myriad of conferences in integrative medicine [5], restorative medicine [6], lifestyle medicine [7], mind-body medicine [8] and nutrition [9] are offering continuing medical education for clinicians. The internet and social media are making consumers/patients more aware of options in addition to medications and surgeries often offered by traditional allopathic healthcare delivery systems. 

In the most recent survey from the National Institutes of Health’s Center for Complementary and Integrative Health on the use of complementary health approaches, several trends are evident. This 2012 survey collected information on the use of various approaches, including dietary supplements, chiropractic or osteopathic manipulation, yoga, massage, and mind-body practices such as meditation. Findings from this survey demonstrated about 33% of adults and 11.4% of children use some form of complementary health approaches. The survey also found that roughly 59 million Americans spend money out-of-pocket on complementary health approaches, with total spending around $30.2 billion/year. Pain was one of the main reasons people sought out complementary therapies, and the most utilized mind-body approaches were chiropractic manipulation, meditation, massage and yoga. Interestingly, yoga has become more popular among children as well in the last 15 years: 3.1% of US children practiced yoga in 2012, compared to 2.3% in 2007. These are samples of a large set of data illustrating the increase in interest, use and willingness to pay for integrative (here described as complementary) therapies [10].

The growing demand and value recognition of such therapies and practices has led many healthcare systems to incorporate integrative medicine’s modalities, practitioners and practices in service to their patients and families. A 2010 survey by Samueli Institute and the American Hospital Association’s Health Forum was conducted to study these trends, entitled Complementary and Alternative Medicine Survey of Hospitals [11]. Out of almost 6000 hospitals receiving the 42-question survey, 714 responded (12%), and 299 of respondents (42%) offered one or more complementary and alternative medicine (CAM) therapies. There was significant regional variation, with the largest number of respondents offering CAM being in the East-North-Central region (23%; Illinois, Indiana, Michigan, Ohio, Wisconsin) and the fewest being in the East-South-Central region (3%; Alabama, Kentucky, Mississippi, Tennessee). Almost half of the respondent institutions offering CAM were academic medical centers, or teaching hospitals, and 72% were urban. The most commonly offered inpatient CAM therapies included pet therapy, massage therapy, music therapy, guided imagery and relaxation training, and Reiki or therapeutic touch. Outpatient modalities were most commonly massage therapy, acupuncture, guided imagery, meditation, relaxation and biofeedback [12].

Despite growing interest and demand for an integrative approach in healthcare, acceptance and adoption of these modalities into mainstream healthcare systems remains limited, and thus inaccessible for many patients and families. Contributing factors to this lack of access include inadequate numbers of trained practitioners, lack of insurance reimbursement for interventions, billing or productivity structures which do not support the time needed to do significant patient counseling and education, and other financial or ideological limitations of healthcare institutions and individual providers alike [13,14]. Medical education at all levels remains limited in training physicians with integrative medicine tools and non-pharmacologic alternatives to symptom management or disease treatment and modification. There are, however, increasing efforts among some medical schools to incorporate lifestyle medicine curricula [15], an integrative medicine elective, or some exposure to CAM therapies in their curriculum. According to the Association of American Medical Colleges, 43% of medical schools offered CAM in their curricula as of 2010. In addition, a survey of University of California medical students’ perceptions about CAM, 75% felt that “western medicine would benefit from incorporation of CAM therapies and ideas” [11]. At the Graduate Medical Education level, programs such as Integrative Medicine in Residency (IMR), an online curriculum, is now utilized in 63 residency programs nationally and internationally at the time of this writing [16]. In 2014 the American Board of Physician Specialties began offering a national integrative medicine board exam, for physicians who have completed a two-year fellowship in integrative medicine [17], adding further recognition and standardization to the field of integrative medicine. 

One common example of a complex, multi-faceted problem seen from primary care to palliative care is anxiety. Children and teens are experiencing anxiety at unprecedented rates, and according to Kessler et al. in a National Comorbidity Survey of adolescents, the lifetime prevalence of any anxiety disorder was 31.9% between the ages of 13–18 years [18], with females (38%) being affected more than males (26%). Although this does not separate adolescents with comorbidities, it is well known that this population group often suffers from anxiety at higher rates [19,20].

Our understanding of anxiety grows by the week, with more studies detailing the connection between gut health and its role in anxiety [21], depression [22,23], pain, obesity, diabetes [24], autoimmune diseases, and the list goes on. Additionally, a history of adverse childhood experiences (ACEs) such as trauma, neglect or abuse predisposes one to an increased risk of developing anxiety over their lifetime [25]. Social media use, sleep deprivation and social isolation have all been associated with higher rates of anxiety [26]. Genetics, diet [27,28], family history and environmental exposures also have significant bearing on an individual’s mental health. Given the numerous etiological influences, and individual variation in life experiences and exposures, it is not reasonable to expect that a single drug or treatment would be adequate for such a multidimensional condition. 

Numerous studies, as well as centuries of tradition in eastern medicine, support the efficacy of mind-body techniques for management of anxiety, chronic pain, illness and stress. Examples include various types of meditation [29]; hypnosis and self-hypnosis [30,31], mindfulness-based stress reduction (MBSR) [32], and yoga [33]. Despite the evidence-supported efficacy and cost-savings, providers are not routinely trained in these treatments over a pharmaceutical approach. Integrative medicine interventions carry a low risk of harm, making them an attractive adjunct or when appropriate, alternative to pharmaceuticals, which are not without adverse risks. An additional benefit of integrative therapies is the active participation required by the patient in the regulation of their own nervous system or mind when addressing problems, allowing the patient to feel in control. These approaches are highly empowering and without significant cost once the technique has been adequately taught to the patient and family to employ when desired. Integrative medicine combines all appropriate therapies using an evidence-based approach with consideration to safety, tolerability and efficacy. Studies have shown that combining mind-body techniques with pharmacologic interventions can reduce distress. For example, pediatric leukemia patients undergoing painful procedures in a study comparing a combined intervention (CI) with pharmacologic only (PO) demonstrated significant reduction in distress associated with the procedure in the CI arm, compared to the PO group [34].

This example illustrates how complex and multifaceted many of today’s medical issues are, and ways in which integrative medicine can address that complexity using a myriad of tools and modalities suited for a variety of settings and preferences. Many such techniques can be employed in the outpatient, inpatient or community health care setting, with little to no cost and are adaptable to a variety of cultures, backgrounds, ages, disease-states and patient preferences. 

## 3. Overview of Integrative Medicine Modalities

Specific domains in which integrative medicine providers offer care, interventions and education for patients may include nutrition, mind-body techniques, regulation of stress physiology, sleep support, therapeutic movement, Reiki and other energy work techniques, use of herbs and supplements, aromatherapy, mental health support and identification/modification of environmental factors contributing to disease. Knowledge of other healing systems, such as traditional Chinese medicine, Ayurveda, naturopathy, chiropractic and homeopathy, allows integrative medicine providers from an allopathic background to appreciate the role these fields play in healing, and appropriately counsel patients seeking care. 

We will briefly describe the following common, well-tolerated integrative modalities that can be used to support patients receiving hospice and palliative care: acupuncture/acupressure, relaxation and stress-physiology regulation techniques, essential oils, botanicals and supplements and reiki.

### 3.1. Acupuncture/Acupressure

Acupuncture is an ancient tradition in which the practitioner inserts thin needles in the body to manipulate specific points. It is a key component of traditional Chinese medicine and is also used for the management of distressing symptoms such as pain and nausea. Acupuncture points can also be stimulated noninvasively with the use of low level lasers or microcurrent. Acupuncture is safe when performed by qualified professionals and is well tolerated by children [35,36]. Acupressure uses firm, steady pressure at these same points. Depending on the location of the acupressure points, and the indications, patients are sometimes able to perform this on themselves. Shonishin is another needleless acupuncture technique that uses pressure and massage along the meridians to influence the flow of energy.

### 3.2. Relaxation Techniques and Stress-Physiology Regulation

The ancient practices of meditation, yoga, tai chi and qi gong are making their way into western culture, as well as being utilized to address a range of present day health problems. In each of these practices, the activation of the parasympathetic nervous system, through intentional, regulated breathing, has been shown to improve autonomic nervous system balance, heart-rate variability, chronic pain, fibromyalgia, anxiety, depression, post-traumatic stress disorder and promote overall well-being [37,38,39,40]. The activation of the parasympathetic nervous system in what has been termed the “relaxation response”, as described above, counters the often overactive sympathetic nervous system stress response, triggered by numerous stimuli that occurs during illness, pain and threatening circumstances. The ability for a person to regulate the stress response and elicit the relaxation response is empowering, and often shifts the locus of control for patients during illness, anxiety, pain and especially at the end-of-life. The use of these physiologic principles in healthcare settings includes modalities such as breathing practices, guided imagery, self-hypnosis and progressive muscle relaxation (PMR). All of these practices have the potential to elicit the relaxation response and can be tailored to individual preferences, functional limitations, age, experiences and culture [41]. 

### 3.3. Essential Oils

Aromatherapy is the use of aerosolized or inhaled essential oils, the potent extracts of roots, plants or herbs, for therapeutic purposes. Topical application of essential oils diluted in a carrier oil, with or without massage, is also a route by which therapeutic effects may be obtained. The parts of the plant used, the species, and the quality of the extract all bear on the quality of the essential oil and thus its therapeutic effect [42].

Essential oils have been used for centuries in many cultures and have also made their way into western healing practices and alongside allopathic medicine in many places. Examples of these uses will follow, related to specific symptoms commonly encountered in patients who are receiving palliative care services.

### 3.4. Botanicals and Supplements

For some patients, due to the burden of polypharmacy, additional supplements are not desired. In others, taking supplements may improve symptoms and allow them to feel a greater sense of self-management over their treatment. Supplements can be taken orally, sublingually or absorbed through the skin. It is critical that any health care provider ask patients about their use of supplements and botanicals, with consideration to any possible side effects as well as drug interactions. An extensive treatment of this topic is beyond the scope of this paper; several specific supplements are covered below in the section on symptom management. 

### 3.5. Reiki

Reiki is an ancient healing art which involves the gentle laying on of hands with the intent to connect the universal life-force energy with the person’s own innate power to heal. This treatment is individualized, can be empowering to the patient, and generally has no adverse effects or harm. 

## 4. Integrative Medicine for Distressing Symptoms in Critical Illness and Palliative Care 

Below we discuss the integrative approach to several common distressing symptoms to those receiving palliative care: nausea and vomiting, pain and neuropathic pain, stress, and dyspnea.

### 4.1. Nausea and Vomiting

Nausea and/or vomiting are common symptoms that develop in serious illness related to a wide range of disorders and/or the treatments used to address them. Traditional pharmacologic treatments, such as ondansetron and metoclopramide, do not come without side effects and limitations. Integrative therapies have been shown to provide significant relief as both primary and adjunct therapies in the management of nausea/vomiting with few side effects or adverse reactions. Herbal supplements, such as oral ginger, have long been regarded as a natural antiemetic, but its demonstrated effectiveness in scientific studies remains inconclusive. Currently, ginger has been used to address nausea/vomiting related to motion sickness, pregnancy, post-surgical and chemotherapy [43,44,45]. In a systematic review of randomized control trials studying the efficacy of ginger for post-operative nausea and vomiting, Ernst et al. concluded that ginger was superior to placebo and demonstrated equal effectiveness compared to metoclopramide [46]. There are few trials related to chemotherapy induced nausea and vomiting, a common problem plaguing many pediatric palliative care patients with cancer. Although the availability of literature yields mixed results, some studies have shown statistically significant benefit. One of the largest randomized control trials to date involved 576 patients undergoing chemotherapy cycles demonstrated a significant decrease in nausea when using ginger compared with placebo [47].

Acupuncture and acupressure have also been utilized to address nausea and vomiting. Specific to pediatrics, there is a rising popularity of acupuncture, with now over one-third of pediatric pain treatment programs offering services [48]. Studies have demonstrated many significant benefits for a myriad of systems, with the strongest evidence demonstrated in the post-operative period. For example, a 2015 systematic review of randomized trials utilizing wrist acupuncture for postoperative nausea and vomiting in both children and adults found a significant reduction in symptoms and need for rescue antiemetics compared to sham treatment [49]. Studies involving exclusively pediatric patients remain limited, although a meta-analysis of clinical studies investigating acupuncture for postoperative nausea/vomiting in children following tonsillectomy further supported these findings [50]. Of note, acupuncture has proven to be well tolerated and safe in the pediatric population. One systematic review involving over 140 randomized clinical trials and 12,000 pediatric patients found acupuncture to be safe for when performed by trained practitioners, with over 90% of adverse events being mild [51].

Aromatherapy, via direct application or inhalation, may also provide benefit in the management of nausea and vomiting. For example, the use of peppermint oil has been recognized as an effective treatment for controlling nausea [52]. Other products, such as ginger oil, have also been utilized in aromatherapy application, with the most updated Cochrane review concluding that participants who received aromatherapy may require less antiemetic medications, although the strength of this evidence was lacking [52]. It is important to state that direct application of peppermint oil, specifically products diluted with methanol, to the nares or chest of infants is not recommended due to the documented risk of apnea, laryngeal and bronchial spasms, and acute respiratory distress [53,54].

### 4.2. Pain

In chronic disease, throughout the continuum of care and at the end-of-life, many patients struggle with uncontrolled pain. The balance between comfort and over sedation can be difficult for caretakers and families. The most common pharmacologic approach to pain management currently relies heavily on opioid medications, which may have unintended side effects. The Support study, which included more than 4000 hospitalized patients at the end-of-life, demonstrated that over 50% of patients suffered from moderate to severe pain in the days before their death [55]. Integrative modalities can be an effective adjunct to traditional pharmacologic approaches to pain.

In recent years, various relaxation techniques in the management of pain have been studied. These include progressive muscle relaxation, guided imagery, and autogenic training, with the goal of inducing one’s own natural relaxation response, described above. For example, a meta-analysis of randomized clinical trials utilizing guided imagery for pain control demonstrated a significant reduction in non-musculoskeletal pain in 11 out of 15 trials reviewed [56]. Studies involving pediatric patients remain limited, but the same principles can be extended to this population with adjustments for age and developmental capabilities. 

Acupuncture has become increasingly recognized in western medicine for the management of both acute and chronic pain, making it a viable adjunct for patients suffering with pain at the end-of-life. One Cochrane review concluded that acupuncture demonstrated effectiveness as a treatment for pain associated with migraines, headaches, neck pain, arthritis, and low back pain, all of which often plague those in the end-of-life [57]. A majority of research specific to the field of hospice and palliative medicine focuses on cancer patients, with many positive results shown with the use of acupuncture not only in pain relief, but also decreased use of traditional analgesics [58,59,60]. For example, one case series of 29 cancer patients at the end-of-life with severe pain demonstrated significant relief and discontinuation of injection analgesics (i.e., morphine, hydromorphone, fentanyl) in 62% of patients, and decreased use in an additional 27% [61].

As one of its many benefits, aromatherapy has been used for pain relief, especially when combined with therapeutic massage, and is another modality to be considered in the management of pain, as well as anxiety and stress. A meta-analysis of literature examining the effectiveness of aromatherapy in treating multiple types of pain found a significant positive effect in reducing pain, typically reported on a visual analog scale [62]. Specific to the pediatric population, one study demonstrated a 40% reduction of acetaminophen usage in children recovering from tonsillectomy who were treated with lavender aromatherapy [63]. Although there are no known studies in the pediatric palliative population, given proven benefits in other population groups, further research of the use of aromatherapy should be pursued. 

### 4.3. Neuropathic Pain

Neuropathic pain can be a significant cause of discomfort and distress to those receiving palliative care and at the end-of-life. Neuropathy can result from multiple etiologies, but the mechanism of pain remains the same, with nerve damage and resultant hyperexcitability of peripheral nerves. Traditional therapy modalities for neuropathic pain, such as neuroleptic drugs, antidepressants, and opioids, have variable success and frequent unwanted side effects. Topical capsaicin is a treatment modality that may be considered for neuropathic pain. Produced from chili peppers, capsaicin binds nociceptors in the skin, leading to initial stimulation and release of substance P. Research has shown that repeat applications of capsaicin can deplete substance P, ultimately resulting in nerve desensitization [64]. Several studies have demonstrated symptom relief in comparison to placebo with the use of topical capsaicin, including Mason et al.’s review of randomized, controlled trials examining the use of capsaicin for chronic neuropathic and musculoskeletal pain in adults [65]. In addition, a recent Cochrane review examined the use of high concentration capsaicin (8% capsaicin patch) for treatment of neuropathic pain, finding moderate to substantial pain relief in patients with several types of neuropathic pain. Additional improvements in sleep, fatigue, depression, and quality of life were also demonstrated. The most common adverse effect to be noted is initial burning at the application site, which decreases with continued use [66].

### 4.4. Stress Response Regulation, Depression, Anxiety and Insomnia

Any clinician caring for a patient who is receiving palliative care must be prepared to address the psychological symptoms common at the end-of-life in addition to the physical ones. Furthermore, the psychological and the physical are physiologically interconnected. Depression and anxiety may plague patients and non-pharmacologic treatments have long been utilized in the management of depression and anxiety. 

Mind-body and relaxation techniques, previously detailed for pain relief, have been shown to provide significant relief of depression, anxiety, and stress. A Cochrane meta-analysis of 15 randomized controlled studies of relaxation techniques with patients diagnosed with depression showed improvement compared to no or minimal traditional treatment. It is important to note, the most significant positive outcome was demonstrated when combined with psychological therapies, such as cognitive behavioral therapy [67]. Furthermore, mindfulness meditation has been demonstrated to provide significant improvements in overall mood and well-being, including stress-reduction, anxiety reduction, alleviating depression, and improved sleep cycles [68]. Specific to pediatrics, one study of 13 adolescents suffering from cancer underwent eight weekly meditation sessions (8 of the 13 enrolled). After completion of the course, investigators analyzed differences in overall mood, depression, sleep, and quality-of-life compared to pre-trial participant responses via questionnaire. Those in the treatment group compared to the control group showed a significant improvement in all areas [69].

As noted, aromatherapy and/or massage have also demonstrated benefit in overall well-being, mood, and sleep patterns. A randomized control trial of aromatherapy massage in the hospice setting found statistically significant improvements in sleep scores and depression reduction [70]. 

Reiki has been shown to have benefits of relaxation, decreased pain perception, anxiety reduction, overall sense of well-being, and quality-of-life in the palliative patient population [71]. Similar to reiki, therapeutic touch has also demonstrated significant benefits [72]. 

### 4.5. Dyspnea

Patients often experience shortness of breath, or dyspnea, related to their underlying disease process and this can worsen during the last six months of life and at the end-of-life. Beyond the physical discomfort, dyspnea can also be anxiety-provoking for many patients and caregivers who have to watch their loved ones suffer. Although the most effective management is to modify the underlying etiology, often in the terminally ill this is not possible. Traditional therapies such as opioids, steroids, nebulizers, and supplemental oxygen can be effective, but not always sufficient [73]. Complementary methods and therapies can provide significant relief as an adjunct therapy. For example, Acupuncture and acupressure have proven beneficial in the chronic-obstructive pulmonary disease (COPD) and asthma patient populations. Jobst et al. conducted a single-blind randomized control trial of 24 COPD patients with disabling shortness of breath who underwent 13 sessions of acupuncture over a three-week period compared to sham acupuncture. Patients in the treatment group reported less subjective breathlessness [74]. Although studies remain limited, and more research is needed in the pediatric population, these benefits could be extended to the palliative care population in pediatrics as well. 

Below we provide a case illustration to demonstrate how integrative medicine can be used in practice to improve quality of life.

## 5. Case Illustration

Janey was a 16-year-old who presented to her general practitioner with complaints of abdominal pain and was found to have angiosarcoma of the spleen with metastasis to the liver, spine and portal venous system. She was being raised by her grandparents due to parental substance abuse. Following the diagnosis, she underwent first line therapy with chemotherapy and radiation. Unfortunately, the social disconnection from her peers created by her medical therapies and the psychological distress of her diagnoses led to a mental health crisis that resulted in a suicide attempt by overdose. While Janey recovered medically in the hospital, the Integrative Medicine team was consulted in the hopes that they might offer a more holistic approach to the impacts of cancer on Janey’s life. 

The integrative medicine practitioner worked intensively with Janey around the use of guided imagery to relieve anxiety and fears related to her diagnosis and uncertain future. Specifically, they chose to focus on scenes in which Janey traveled to Bora Bora, which she had seen in a travel magazine and viewed as the most relaxing location she could imagine. This technique combined with diaphragmatic breathing and aromatherapy was particularly helpful during times of heightened anxiety when waiting for results of imaging studies to evaluate the effectiveness of her ongoing treatment. 

Computed tomography (CT) scans early in the second year of her treatment showed progression of the hepatic portion of her cancer. Discussions about the possibility that she may not be cured became more frequent with her medical team. While she proceeded with a new chemotherapy regimen, she also began planning a Make-A-Wish trip to Bora Bora. Planning her trip motivated her to intensify her practice of integrative strategies and she began a daily meditation practice and engaged in biofeedback. With minimal medications, she had excellent symptom management and was able to enjoy the trip and add more depth to her subsequent imagery work.

By the third year of her cancer journey, her tumor was again growing more quickly, and Janey struggled with progressive abdominal pain. She was very reluctant to use opioid pain medications due to fears about addiction raised by her parents’ substance abuse histories. She was motivated to maintain as normal a life as possible despite her medical condition and prioritized having a job babysitting for the neighbors’ children each afternoon. Her palliative care physician and integrative medicine practitioner worked with her to design a plan involving long- and short-acting pain medications, as well as mind-body practices, to enable her to maintain function, acceptable pain control and still attend to her responsibilities and relationships. 

This plan was quite effective for many months before the cancer progressed. At that time, she had processed many of her fears about addiction with psychology support and was able to accept increased medication for pain control when integrative techniques alone could not manage her symptoms. Janey was aware that she was approaching the end of her life and worked with a child life specialist to participate in legacy activities. 

Janey spent many weeks planning for a wedding to her longtime boyfriend and utilized her arsenal of imagery, breathing, aromatherapy and techniques learned in biofeedback to minimize medications used during the ceremony and reception. As her liver failure progressed, nausea became a prominent symptom and she sought new strategies for treatment. She found a combination of lorazepam and acupressure wrist bands with peppermint aromatherapy and imagery to be most effective. Near the end of her life, she became minimally wakeful and less able to utilize strategies herself. However, her husband and grandparents were able to perform massage with her lavender essential oil and use home music therapy provided through hospice to keep her relaxed as she died. Family participation in integrative strategies made loved ones feel empowered to help Janey through the last stage of her life and has provided crucial legacy memories to help them through their grief.

This case illustrates the numerous ways that integrative medicine modalities were employed alongside, and at times in place of, pharmaceutical and conventional treatments, equipping Janey and her family to manage her disease and symptoms. The shared locus of control (both internal and external) concept is also demonstrated in the combination of integrative therapies as well as medication management, which provided Janey with the ability to live out her life in meaningful ways. 

## 6. Summary

Integrative medicine is the multimodal approach to improved quality of life and patient care that respects and builds upon the strengths of the patient in combination with appropriate medical management. In the setting of palliative and end-of-life care an integrative approach can serve to reduce symptom burden and empower patients and their families.
